# LAPTM5 Restricts HIV-1 Infection in Dendritic Cells and Is Counteracted by Vpr

**DOI:** 10.1128/spectrum.01382-21

**Published:** 2022-02-28

**Authors:** Jiayue Ouyang, Ying Xiong, Hong Shang, Guoxin Liang

**Affiliations:** a Key Laboratory of AIDS Immunology of Ministry of Health, Department of Laboratory Medicine, the First Affiliated Hospital, China Medical University, Shenyang, China; b Research Institute for Cancer Therapy, the First Affiliated Hospital, China Medical University, Shenyang, China; c National Clinical Research Center for Laboratory Medicine, the First Affiliated Hospital of China Medical University, Shenyang, China; Fundacio irsiCaixa

**Keywords:** HIV, host restriction factor, LAPTM5, dendritic cells

## LETTER

Vpr is a viral accessory protein in primate lentiviruses, including human immunodeficiency virus 1 (HIV-1), HIV-2, and simian immunodeficiency virus, and is required for high viral load and disease progression *in vivo* (1, 2). Vpr also enhances HIV-1 replication in primary macrophages and dendritic cells. Similarly, as Vpr increases viral envelope protein (Env) levels in primary macrophages (3), Vpr has also been reported to promote HIV-1 infection in dendritic cells by enhancing Env levels (4). Recently, we have demonstrated that lysosomal-associated transmembrane protein 5 (LAPTM5) is responsible for the long-sought anti-HIV-1 host restriction factor in primary macrophages suppressed by Vpr (5). We also observed high LAPTM5 expression levels in monocyte-derived dendritic cells (MDDCs), prompting us to investigate the relationship between Vpr and LAPTM5 in MDDCs and explore whether Vpr also counteracts the restriction of LAPTM5 to promote HIV-1 infection in dendritic cells.

**FIG 1:**
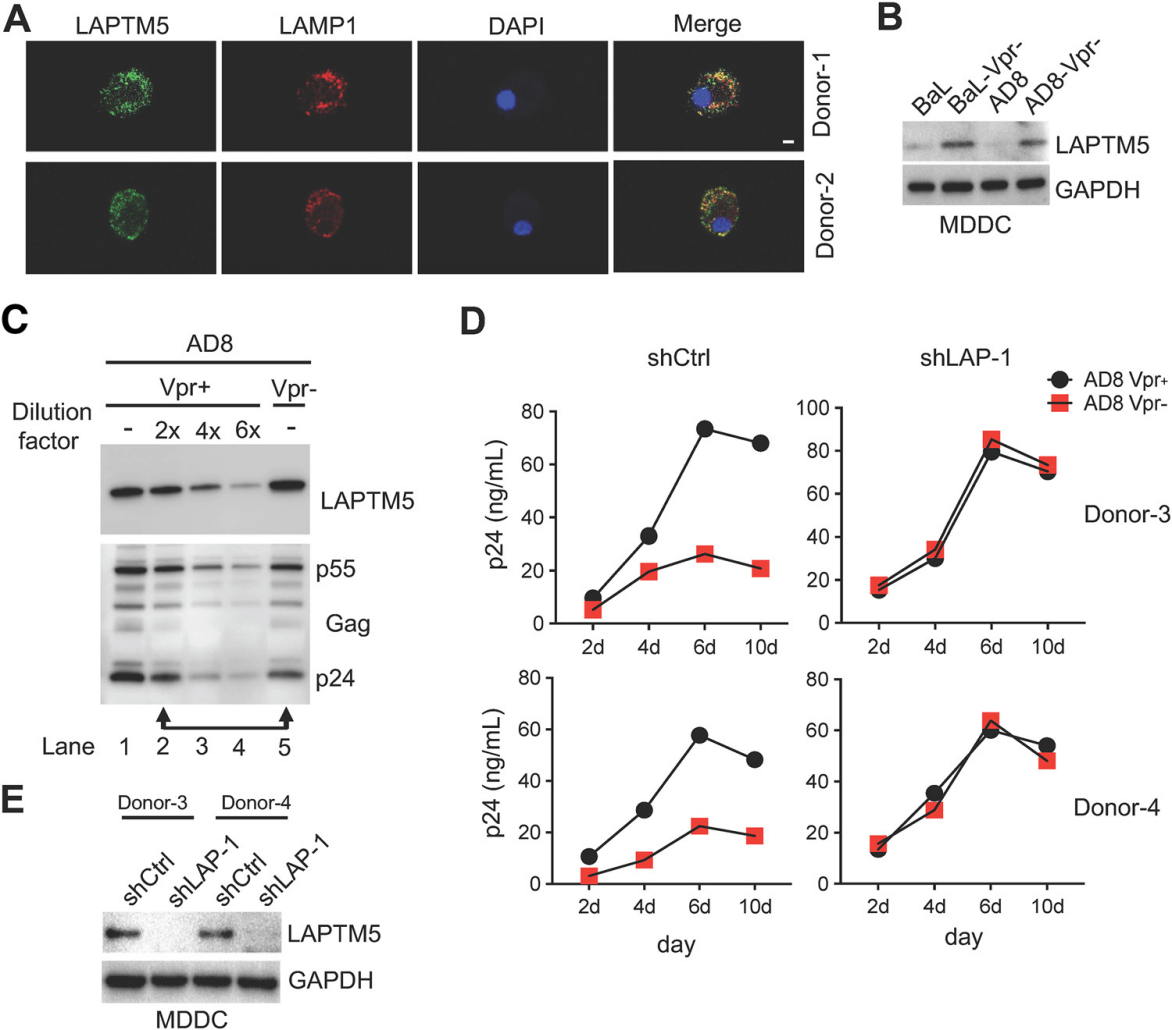
Vpr overcomes LAPTM5 to promote HIV-1 infection in dendritic cells. (A) Monocyte-derived dendritic cells (MDDCs) were fixed and immunostained with anti-lysosomal-associated transmembrane protein 5 (LAPTM5) or anti-lysosomal marker protein (LAMP1) antibodies and probed with a secondary antibody conjugated with Alexa Fluor 488 or 555. Cells were stained with 4′,6-diamidino-2-phenylindole (DAPI); scale bar, 10 μm. (B) MDDCs were infected with 100 ng of wild-type or Vpr-defective HIV-1 for 8 days. Cells were lysed for Western blotting to assess LAPTM5 and GAPDH expression. (C) MDDCs infected with 100 ng of replication-competent wild-type or Vpr-defective HIV-1. Cell lysates from wild-type HIV-1 were diluted as indicated for Western blotting to assess LAPTM5 and Gag by their specific antibodies. (D and E) Lentiviral short hairpin RNA (shRNA)-transduced MDDCs were treated with VLP-Vpx and infected with 100 ng of wild-type or Vpr-defective HIV-1_AD8_ for 10 days. A P24 enzyme-linked immunosorbent assay (ELISA) was used to measure viral production at the indicated time points (D). Before the infection, the aliquoted shRNA-transduced cells were lysed for Western blotting to assess LAPTM5 and GAPDH expression (E).

At the beginning, we determined the expression of LAPTM5 and its subcellular localization in MDDCs generated from two independent healthy donors. MDDCs were fixed and stained with specific antibodies against LAPTM5 or the lysosomal marker protein lysosomal-associated membrane protein 1 (LAMP1). LAPTM5 was consistently colocalized with LAMP1 protein in the lysosome (Fig. 1A), suggesting that LAPTM5 also implements its function in lysosomes in MDDCs. Afterward, we were interested in whether Vpr could promote LAPTM5 degradation in MDDCs. We subsequently infected MDDCs with wild-type HIV-1 and its Vpr-defective counterpart for 8 days and observed that the presence of Vpr indeed resulted in LAPTM5 degradation (Fig. 1B). Since wild-type HIV-1 infection could result in a higher level of infection efficiency in MDDCs, we next analyzed the level of LAPTM5 protein in the similar infected cells. As indicated by the arrow, LAPTM5 protein was also reduced by wild-type HIV-1 in contrast to Vpr-defective HIV with similar levels of infection (Fig. 1C; lane 2 versus lane 5). Taken together, the results indicate that Vpr could promote LAPTM5 degradation. This result suggests that Vpr degrades LAPTM5 in MDDCs as it does in primary macrophages, indicating the possibility that Vpr also promotes LAPTM5 degradation to enhance HIV-1 infection in dendritic cells.

To confirm this hypothesis, we investigated a functional analysis for endogenous LAPTM5 and Vpr in primary MDDCs. We used CCR-5 trophic HIV_AD8_ and its Vpr-defective counterpart to infect MDDCs in the presence or absence of LAPTM5. We then used virion-like particles (VLP)-Vpx to deplete host restriction factor SAMHD1 in MDDCs to enhance HIV-1 replication, because MDDCs express a higher level of SAMHD1, which inhibits HIV-1 reverse transcription. We observed striking differences in virion production by MDDCs infected with wild-type and Vpr-defective HIV-1 in the presence of LAPTM5 (Fig. 1D and E), when the virus was allowed to spread through the culture for 10 days. Alternatively, in the absence of LAPTM5, Vpr had no obvious effect on virion production in MDDCs. Therefore, Vpr also counteracts the restriction of LAPTM5 to enhance HIV-1 infection in dendritic cells. Here, we demonstrated that LAPTM5 is also a host restriction factor in dendritic cells.
